# Rise in Use of Digital Mental Health Tools and Technologies in the United States During the COVID-19 Pandemic: Survey Study

**DOI:** 10.2196/26994

**Published:** 2021-04-16

**Authors:** Dara H Sorkin, Emily A Janio, Elizabeth V Eikey, Margaret Schneider, Katelyn Davis, Stephen M Schueller, Nicole A Stadnick, Kai Zheng, Martha Neary, David Safani, Dana B Mukamel

**Affiliations:** 1 Department of Medicine University of California, Irvine Irvine, CA United States; 2 Herbert Wertheim School of Public Health and Human Longevity Science University of California, San Diego San Diego, CA United States; 3 The Design Lab University of California, San Diego San Diego, CA United States; 4 Department of Public Health University of California, Irvine Irvine, CA United States; 5 Department of Pathology University of California, San Francisco San Francisco, CA United States; 6 Department of Psychological Science University of California, Irvine Irvine, CA United States; 7 Department of Informatics University of California, Irvine Irvine, CA United States; 8 Department of Psychiatry University of California, San Diego San Diego, CA United States; 9 UC San Diego Dissemination and Implementation Science Center San Diego, CA United States; 10 Child and Adolescent Services Research Center San Diego, CA United States; 11 Department of Psychiatry University of California, Irvine Irvine, CA United States

**Keywords:** COVID-19, digital technologies, mHealth, mental health, anxiety, depression, MTurk, e-mental health, digital health, distress, self-management

## Abstract

**Background:**

Accompanying the rising rates of reported mental distress during the COVID-19 pandemic has been a reported increase in the use of digital technologies to manage health generally, and mental health more specifically.

**Objective:**

The objective of this study was to systematically examine whether there was a COVID-19 pandemic–related increase in the self-reported use of digital mental health tools and other technologies to manage mental health.

**Methods:**

We analyzed results from a survey of 5907 individuals in the United States using Amazon Mechanical Turk (MTurk); the survey was administered during 4 week-long periods in 2020 and survey respondents were from all 50 states and Washington DC. The first set of analyses employed two different logistic regression models to estimate the likelihood of having symptoms indicative of clinical depression and anxiety, respectively, as a function of the rate of COVID-19 cases per 10 people and survey time point. The second set employed seven different logistic regression models to estimate the likelihood of using seven different types of digital mental health tools and other technologies to manage one’s mental health, as a function of symptoms indicative of clinical depression and anxiety, rate of COVID-19 cases per 10 people, and survey time point. These models also examined potential interactions between symptoms of clinical depression and anxiety, respectively, and rate of COVID-19 cases. All models controlled for respondent sociodemographic characteristics and state fixed effects.

**Results:**

Higher COVID-19 case rates were associated with a significantly greater likelihood of reporting symptoms of depression (odds ratio [OR] 2.06, 95% CI 1.27-3.35), but not anxiety (OR 1.21, 95% CI 0.77-1.88). Survey time point, a proxy for time, was associated with a greater likelihood of reporting clinically meaningful symptoms of depression and anxiety (OR 1.19, 95% CI 1.12-1.27 and OR 1.12, 95% CI 1.05-1.19, respectively). Reported symptoms of depression and anxiety were associated with a greater likelihood of using each type of technology. Higher COVID-19 case rates were associated with a significantly greater likelihood of using mental health forums, websites, or apps (OR 2.70, 95% CI 1.49-4.88), and other health forums, websites, or apps (OR 2.60, 95% CI 1.55-4.34). Time was associated with increased odds of reported use of mental health forums, websites, or apps (OR 1.20, 95% CI 1.11-1.30), phone-based or text-based crisis lines (OR 1.20, 95% CI 1.10-1.31), and online, computer, or console gaming/video gaming (OR 1.12, 95% CI 1.05-1.19). Interactions between COVID-19 case rate and mental health symptoms were not significantly associated with any of the technology types.

**Conclusions:**

Findings suggested increased use of digital mental health tools and other technologies over time during the early stages of the COVID-19 pandemic. As such, additional effort is urgently needed to consider the quality of these products, either by ensuring users have access to evidence-based and evidence-informed technologies and/or by providing them with the skills to make informed decisions around their potential efficacy.

## Introduction

### Background

On March 11, 2020, the World Health Organization designated the COVID-19 outbreak a global pandemic, which, among other things, has led to unprecedented hazards to mental health globally [1]. Individual states within the United States began to implement measures to contain the spread of the virus, including limiting travel, mandating physical distancing, and limiting nonessential medical visits. By April 2020, nearly 200,000 cases of COVID-19 and more than 5000 deaths had been reported in the United States [2].

In addition to the unpredictability and uncertainty of the pandemic itself, policy efforts to mitigate risk, such as stay-at-home orders and/or social distancing, introduced a number of additional stressors including social isolation, inactivity, loss of income, and lack of access to basic services, to name but a few [3]. This may be why, mirroring the increase in COVID-19 cases and deaths, there has been an increase in mental distress [1,4-6]. For example, data from the US Census Bureau reported that adults assessed as part of a nationally representative survey in April and May 2020 were more than three times as likely to screen positive for depressive disorders, anxiety disorders, or both, relative to a comparable sample in 2019 [7]. In a similar vein, the Centers for Disease Control and Prevention reported significantly elevated levels of adverse mental health conditions, substance use, and suicidal ideation resulting from the COVID-19 pandemic, with these mental health conditions disproportionately affecting specific populations, such as young adults, Hispanic persons, Black persons, essential workers, unpaid caregivers of adults, and those receiving treatment for preexisting psychiatric conditions [8].

As medical organizations and hospital systems, including those that address mental health, quickly pivoted to digital platforms such as videoconferencing/teleconferencing and patient-provider SMS text messaging [9], the documented growth in and reliance on eHealth/telehealth use emerged as a viable solution to continue providing health and mental health services [10-12]. Indeed, it has been suggested that the COVID-19 pandemic is serving as a “black swan” moment for mental health care—an unforeseen event that will permanently shift mental health care provision toward online prevention, treatment, and care [12]. Given the increase in mental health issues resulting from the pandemic, digital platforms also offer the potential to provide scalable, nonconsumable mental health resources, as they may be less reliant on trained providers [13]. A nonconsumable treatment is one that, once used, retains its therapeutic potential. Unlike a dose of medication, which will not benefit another person once used, digital resources can be used by many people and continue to be helpful [14].

In tandem with this increase in the use of telehealth services and digital platforms, technology companies have also reported increased demand for digital mental health products and therapeutics since the start of the COVID-19 pandemic [15]. However, there have been no empirical studies examining rates of use of these digital mental health products and therapeutics during the COVID-19 pandemic. Examination of marketplace trends through app analytics platforms (eg, App Annie) indicate that downloads and engagement have increased since the onset of COVID-19 [16]. However, it is unclear if reported rates of growth relate to the pandemic or represent an already documented gradual trend of engagement [17]. In light of the transformative changes that have occurred in the delivery of health and mental health care resulting from the COVID-19 pandemic, understanding potential changes in consumer behavior pertaining to the use of digital mental health tools and other technologies during the COVID-19 pandemic is needed.

### Current Study

To this end, this study examined the following questions:

1. To what extent was the likelihood of having symptoms indicative of clinical depression and anxiety associated with the county COVID-19 case rate and time?
2. Were individuals with moderate to high self-reported depressive or anxious symptomatology more likely to use digital mental health tools and other technologies compared to individuals who endorsed low depression or anxiety symptom experiences?
3. If so, did the differences in use of these digital mental health tools and other technologies among those with high versus low symptom endorsement vary according to the COVID-19 case rate?

## Methods

### Participants and Procedures

Participants were recruited from Amazon Mechanical Turk (MTurk), an online crowdsourcing platform commonly used in behavioral science studies [18]. Survey data collection occurred across all 50 states and Washington DC during 4 one-week periods in April, May, June, and July 2020, starting on approximately the 6th of each month. At the first time point, we aimed to recruit approximately 1250 people. At each of the following time points, we aimed to recruit approximately 1750 people.

MTurk allows researchers to collect a large amount of quality data quickly and for relatively little cost [19-21]. On MTurk, requesters are people who post or request tasks (eg, surveys) to be completed, whereas workers are people who are paid for task completion. Requesters can customize the tasks to be available to certain MTurk workers. MTurk workers are able to read descriptions of tasks and select the tasks they are interested in.

For the purpose of this study, study participants had to meet three eligibility criteria: (1) be at least 18 years old, (2) currently reside in the United States, and (3) not have completed the survey in a prior wave. If eligible, participants completed the survey via Qualtrics and received US $6 via MTurk as compensation upon completion. The protocol was approved by the Institutional Review Board (IRB#2019-5406) at the University of California, Irvine.

### Measures

#### Symptoms Indicative of Clinical Depression and Anxiety

The Patient Health Questionnaire-9 (PHQ-9) [22] is a self-report measure used to assess depressive symptoms. It consists of the nine criteria upon which the diagnosis of major depressive disorder is based, according to the Diagnostic and Statistical Manual of Mental Disorders (DSM-IV). The questionnaire uses a 4-point Likert scale (0=not at all, 1=several days, 2=more than half the days, 3=nearly every day) to gauge responses to questions about participants’ mental health over the previous 2-week period. Scores on the PHQ-9 can range from 0-27, with 0-4 suggesting no depression symptoms, 5-9 mild symptoms, 10-14 moderate symptoms, 15-19 moderately severe symptoms, and 20-27 more severe depression [23], with a cut-off score of 10 or above being used in this study to indicate the presence of clinically elevated levels of depression symptoms (0=minimal or mild depressive symptomatology and 1=moderate to severe depressive symptomatology). This cut-off has been shown to have high sensitivity (88%) and specificity (88%) [22]. In the present sample, reliability was strong (unweighted α=.92).

The Generalized Anxiety Disorder-7 Questionnaire (GAD-7) [24] was used to assess generalized anxiety disorder. Respondents rate answers (0=not at all, 1=several days, 2=more than half the days, 3=nearly every day) to 7 questions assessing anxiety symptoms experienced over the past two weeks, with the total score ranging from 0-21; scores from 0-4 suggest minimal symptoms, 5-9 mild symptoms, 10-14 moderate symptoms, and 15-21 severe symptoms. A cut-off score of 10 or above was used in this study to indicate the presence of elevated levels of anxiety symptoms (0=minimal to mild anxiety symptomatology and 1=moderate to severe anxiety symptomatology). This cut-off has high sensitivity (89%) and specificity (82%) [24]. Reliability in the present study was high (unweighted α=.92).

#### Digital Mental Health Tools and Other Technologies to Support Mental Health

Participants were asked how frequently (never, rarely, sometimes, often, or always) they used 20 different types of technology in the last 7 days to manage or support their mental health. Responses were dichotomized (0=never, rarely, sometimes and 1=often or always). The 20 technology types included the following: (1) mental health online forums or communities (eg, Mental Health Forum, BeyondBlue), (2) mental health websites (eg, NAMI, StudentsAgainstDepression.org), (3) mental health apps (eg, 7 Cups, Headspace, Moodpath), (4) phone-based or text-based crisis lines (eg, Crisis Text Line, Suicide Prevention Lifeline), (5) other health online forums or communities (eg, MyFitnessPal forum), (6) other health websites (eg, WebMD, WHO), and (7) other health apps (eg, LoseIt, MapMyRun, Sleep Cycle, Flo). Additionally, participants were also asked about their use of the following technologies to manage their mental health: (8) social media (eg, Facebook, Instagram, Twitter, Snapchat), (9) blogs, (10) online, computer, or console gaming/video gaming, (11) online calendar, checklist, or planner, (12) Word, Notepad, or Google Docs, (13) spreadsheet or Google Sheets, (14) email, (15) texting or messaging software, and (16) video conferencing software. Participants were also able to select the following options: (17) other general online forums or communities, which may include mental health communities (eg, Reddit); and writing in (18) other types of websites, (19) other apps, and (20) other types of technologies not listed [25].

The technologies were collapsed into seven categories by theme: (1) mental health forums, websites, or apps, (2) phone-based or text-based crisis lines, (3) other health forums, websites, or apps, (4) social media and blogs, (5) online, computer, or console gaming/video gaming, (6) online calendar, checklist, planner, Word, Notepad, Google Docs, spreadsheet, or Google Sheets, and (7) email, texting or messaging software, or video conferencing software [25].

#### County COVID-19 Case Rate

The COVID-19 Data Repository maintained by the Center for Systems Sciences and Engineering at Johns Hopkins University has provided the COVID-19 case count of each county in the United States on a daily basis since January 22, 2020 [2]. Using participants’ zip codes, the case count in each participant’s county on the date each participant began the survey was merged with the data collected from MTurk. The county case count was converted into the rate of cases per 10 people by dividing the count by each county’s total population and multiplying this number by 10. Total county population was obtained from the US Census Bureau’s American Community Survey 2018 5-year estimates [26].

#### Survey Time Period

Survey response windows that reflected the states’ population sizes were made available during 4 week-long periods in April, May, June, and July of 2020. As such, this variable also serves as a proxy indicator of time. The time point variable was treated as a continuous variable in the analyses.

#### Covariates

Additional variables were included as covariates. These encompassed standard demographic characteristics, including age, sex (1=male, 2=female), race/ethnicity (1=non-Hispanic White, 2=Latino, 3=non-Hispanic Asian, 4=non-Hispanic Black or African American, 5=other), marital status (1=married or living with a partner, 2=single or not living with a partner, 3=separated, divorced, or widowed), employment status (1=no change in employment status due to COVID-19, 2=reduced hours due to COVID-19, 3=lost job due to COVID-19), income level, and education level (1=completed high school or less, 2=some college or more). Covariates also included a variable representing state fixed effects.

### Analytic Sample

A total of 6704 survey responses were collected. After omitting ineligible people due to duplicate or missing MTurk worker identification numbers, the total sample included 5907 participants. Of this group, 2.22% had missing data for at least one question from the PHQ-9 and 1.64% had missing data for at least one question from the GAD-7. Participants’ average PHQ-9 and GAD-7 scores were imputed if at least 50% of the scale questions were answered. Less than 5.51% of sociodemographic data was missing. Binary and continuous variables underwent mean imputation. An additional category for missing data was created for all other sociodemographic covariates. Where participants had a missing zip code, but provided their state of residence, the average county case count and the average county population in the participant’s state were used to calculate the county case rate per 10 people. After mean imputation was executed and missing county case rates were replaced with average rates, the analytic sample included 5899 individuals.

### Statistical Analyses

Summary statistics were calculated to describe the sociodemographic characteristics of and prevalence of anxiety and depression symptoms in the sample. The use of digital mental health tools and other technologies in the sample was also described. Means, standard errors, and proportions were reported.

To examine the extent to which the likelihood of having symptoms indicative of clinical depression and anxiety was associated with the county COVID-19 case rate and time, we estimated two separate logistic regression models, one for each mental health outcome. Adjusted models included age, sex, race/ethnicity, marital status, employment status, income level, education level, and state fixed effects.

To evaluate whether individuals with moderate to high self-reported depressive or anxious symptomatology were more likely to use digital mental health tools and other technologies compared to individuals who endorsed low depression or anxiety symptom experience, we estimated seven different logistic regression models, one for each of the technology types defined above. Symptoms of anxiety, symptoms of depression, county case rate per 10 people, and time point were the independent variables. Adjusted models included age, sex, race/ethnicity, marital status, education level, income level, and state fixed effects. To descriptively assess the use of digital mental health tools and other technologies over time, the predicted probabilities of employing each type of tool or technology were plotted at each time point, while holding the covariates at their mean values. We also plotted the total number of respondents using each type of technology at each time point.

Finally, to understand if differences in use among those with high versus low symptom endorsement varied according to the rate of COVID-19 cases, we further tested the inclusion of two interactions examining the following in these adjusted models: (1) county-level COVID-19 case rate with depression and (2) county-level COVID-19 case rate with anxiety.

Probability weights were generated to account for oversampling and undersampling of participants in each state. Use of probability weights allowed for the proportion of participants from each state to reflect the proportion of the US population that each state population comprises. Weights were generated using data from the American Community Survey 2018 5-year estimates of US state populations. Survey weights were employed when estimating summary statistics and multivariable regression. Additionally, a Šidák-corrected cut-off *P* value (*P*<.0037) was employed to identify significant findings and account for the increased type I error rate that resulted from the use of multiple models [27]. All analyses were conducted in Stata 16 (StataCorp LLC) [28].

## Results

Table 1 shows the weighted means and standard errors for continuous variables, as well as the weighted proportions of individuals within each categorical variable group. Overall, the average age was 36.99, and most of the sample was male, non-Hispanic White, and married or living in a marital-like relationship. Most of the sample did not experience a change in employment status due to the COVID-19 pandemic, had an income level greater than US $50,000, and had greater than a high school education. Close to half of the sample experienced symptoms indicative of clinical levels of depression (45.45%) or anxiety (40.04%).

**Table 1:** Characteristics of analytic sample (N=5899).

Sociodemographic characteristics			Values^a^
County case rate per 10 people, mean (SE)			0.069 (0.0023)
Age, mean (SE)			36.99 (0.17)
Symptoms of depression, %			45.45
Symptoms of anxiety, %			40.04
**Sex, %**			
	Male	57.58	
	Female	42.42	
**Race/ethnicity, %**			
	Non-Hispanic White	61.07	
	Latino	11.69	
	Asian	6.54	
	Black/African American	7.85	
	Other	12.85	
**Marital status, %**			
	Married/living in a marital-like relationship	62.20	
	Single/never married	31.76	
	Separated, divorced, or widowed	6.04	
**Employment status, %**			
	No change in employment status due to COVID-19	64.42	
	Reduced hours due to COVID-19	26.88	
	Lost job due to COVID-19	8.70	
**Income level (US $), %**			
	0-10,000	4.67	
	10,001-20,000	7.42	
	20,001-30,000	11.71	
	30,001-40,000	11.91	
	40,001-50,000	11.85	
	50,001-60,000	12.14	
	60,001-70,000	8.75	
	70,001-80,000	8.60	
	80,001-90,000	5.24	
	90,001-100,000	6.14	
	>100,000	11.56	
**Education level, %**			
	High school or less	19.35	
	More than high school	80.65	
**Use of digital mental health tools and other technologies to manage mental health, %**			
	Mental health forums, websites, or apps	27.39	
	Phone-based or text-based crisis lines	17.74	
	Other health forums, websites, or apps	34.48	
	Social media or blogs	47.73	
	Online, computer, or console gaming/video gaming	33.55	
	Online calendar, checklist, planner, Word, Notepad, Google Docs, spreadsheet, or Google Sheets	44.55	
	Email, SMS texting or messaging software, or video conferencing software	55.50	

^a^The proportion of missing data ranged from 0.0%-5.51%.

Table 2 shows the results of the two adjusted models regressing the prevalence of clinically meaningful symptoms of depression and anxiety, respectively, on the county COVID-19 case rate per 10 people, time point, and the covariates. As shown, COVID-19 case rate was significantly associated with an increase in the likelihood of experiencing clinically meaningful symptoms of depression (odds ratio [OR] 2.06, 95% CI 1.27-3.35), but not anxiety (OR 1.21, 95% CI 0.77-1.88). Additionally, the likelihood of experiencing symptoms of depression or symptoms of anxiety increased over time (OR 1.19, 95% CI 1.12-1.27 and OR 1.12, 95% CI 1.05-1.19, respectively). Results associated with each covariate are shown in Multimedia Appendix 1.

**Table 2:** Association between symptoms indicative of clinical levels of depression and anxiety and rates of COVID-19 cases and time: estimates based on two separate logistic models (N=5899)^a^.

Variables	Model 1: Symptoms of depression, odds ratio (95% CI)	Model 2: Symptoms of anxiety, odds ratio (95% CI)
County-level COVID-19 case rate per 10 people	2.06^b^ (1.27-3.35)	1.21 (0.77-1.88)
Survey time point	1.19^c^ (1.12-1.27)	1.12^c^ (1.05-1.19)

^a^Models adjusted for the following covariates: age, sex, race/ethnicity, marital status, employment status, income level, education level, and fixed state effects.

^b^*P*<.0037 (Šidák-corrected *P* value).

^c^*P*<.001.

The adjusted association between depression, anxiety, county case rate per 10 people, time point, and use of each of the seven categories of digital mental health tools and other technologies is presented in Table 3. Results pertaining to each covariate are shown in Multimedia Appendix 1. Both symptoms of depression and anxiety were significantly associated with the likelihood of using each type of tool and technology. Those with depressive symptoms were three to six times more likely to use digital mental health tools than those without depressive symptoms. Those with symptoms of anxiety were two to three times more likely to use digital mental health tools than those without symptoms of anxiety. Specifically, those with depressive symptoms or symptoms of anxiety were more likely than those without symptoms to use mental health forums, websites, or apps (OR 6.01, 95% CI 4.70-7.70 and OR 2.95, 95% CI 2.37-3.66, respectively), phone-based or text-based crisis lines (OR 4.98, 95% CI 3.66-6.77 and OR 2.85, 95% CI 2.22-3.66, respectively), and other health forums, websites, or apps (OR 3.44, 95% CI 2.81-4.20 and OR 2.55, 95% CI 2.11-3.10, respectively).

The findings pertaining to digital mental health tools mirrored the results from models assessing use of other technologies not necessarily related to health. Specifically, those with symptoms indicative of depression and anxiety were more likely, as compared to those without symptoms, to engage in use of social media and blogs (OR 1.56, 95% CI 1.31-1.86 and OR 1.80, 95% CI 1.51-2.14, respectively); online, computer, or console gaming/video gaming (OR 1.63, 95% CI 1.36-1.95 and OR 1.67, 95% CI 1.40-1.99, respectively); online calendar, checklist, planner, Word, Notepad, Google Docs, spreadsheet, or Google Sheets (OR 1.91, 95% CI 1.60-2.28 and OR 2.09, 95% CI 1.75-2.50, respectively); and email, texting or messaging software, or video conferencing software (OR 1.66, 95% CI 1.39-1.98 and OR 1.82, 95% CI 1.52-2.17, respectively). However, in general, even though significant, the odds ratios for these other technologies tended to be much smaller, with the largest being 2.09 and all others below 2.00, while all the odds ratios for the mental health technologies were well above 2.00, ranging from 2.55 to 6.01.

With regard to the association between county-level COVID-19 case rate and digital mental health tool and other technology use, a higher county case rate per 10 people was associated with increased likelihood of using digital mental health tools, specifically mental health forums, websites, or apps (OR 2.70, 95% CI 1.49-4.88) and other health forums, websites, or apps (OR 2.60, 95% CI 1.55-4.34). Additionally, over time, there was an increase in the likelihood of using mental health forums, websites, or apps (OR 1.20, 95% CI 1.11-1.30); phone-based or text-based crisis lines (OR 1.20, 95% CI 1.10-1.31); other health forums, websites, or apps (OR 1.12, 95% CI 1.05-1.20); and online, computer, or console gaming/video gaming (OR 1.12, 95% CI 1.05-1.19). The predicted probabilities of using each type of technology at each time point while holding the covariates at their mean values are graphed in Multimedia Appendix 2. Multimedia Appendix 3 shows the total number of respondents using each type of technology at each time point.

**Table 3:** Associations between use of digital mental health tools and other technologies and prevalence of mental illness symptoms and the rate of COVID-19 cases: estimates based on seven separate logistic models^a^.

Variables	Model 1: Mental health forums, websites, or apps (n=5849)	Model 2: Phone-based or text-based crisis lines (n=5831)	Model 3: Other health forums, websites, or apps (n=5854)	Model 4: Social media and blogs (n=5788)	Model 5: Online, computer, or console gaming/video gaming (n=5866)	Model 6: Online calendar, checklist, planner, Word, Notepad, Google Docs, spreadsheet, or Google Sheets (n=5835)	Model 7: Email, texting or messaging software, or video conferencing software (n=5849)
	OR^b^ (95% CI)	OR (95% CI)	OR (95% CI)	OR (95% CI)	OR (95% CI)	OR (95% CI)	OR (95% CI)
Symptoms of depression	6.01^c^ (4.70-7.70)	4.98^c^ (3.66-6.77)	3.44^c^ (2.81-4.20)	1.56^c^ (1.31-1.86)	1.63^c^ (1.36-1.95)	1.91^c^ (1.60-2.28)	1.66^c^ (1.39-1.98)
Symptoms of anxiety	2.95^c^ (2.37-3.66)	2.85^c^ (2.22-3.66)	2.55^c^ (2.11-3.10)	1.80^c^ (1.51-2.14)	1.67^c^ (1.40-1.99)	2.09^c^ (1.75-2.50)	1.82^c^ (1.52-2.17)
County-level COVID-19 case rate per 10 people	2.70^d^ (1.49-4.88)	1.81^e^ (1.02-3.19)	2.60^c^ (1.55-4.34)	1.49 (0.95-2.36)	1.65^e^ (1.07-2.56)	2.04^e^ (1.26-3.30)	1.77^e^ (1.09-2.89)
Survey time point	1.20^c^ (1.11-1.30)	1.20^c^ (1.10-1.31)	1.12^d^ (1.05-1.20)	1.08^e^ (1.02-1.15)	1.12^c^ (1.05-1.19)	1.08^e^ (1.01-1.15)	1.08^e^ (1.02-1.14)

^a^Models adjusted for the following covariates: age, sex, race/ethnicity, marital status, employment status, income level, education level, and fixed state effects.

^b^OR: odds ratio.

^c^*P*<.001.

^d^*P*<.0037 (Šidák-corrected *P* value).

^e^*P*<.05.

Lastly, models assessing the interaction of county-level COVID-19 case rate and symptoms indicative of anxiety or depression did not yield significant findings (analyses available on request). For this reason, interaction terms were not subject to further analyses.

## Discussion

To our knowledge, this is the first study to examine rates of use of digital mental health tools and other technologies to manage one’s mental health among a sample of people during the early stages of the COVID-19 pandemic. Similar to other studies [29], rates of COVID-19 cases were associated with increased rates of depressive symptoms. Both depressive and anxiety symptoms and COVID-19 county case rates were associated with the largest increased likelihood of using digital mental health tools, specifically mental health forums, websites, and apps; phone-based/text-based crisis lines; and other health forums, websites, and apps, reflecting the greatest increase in likelihood of use for products designed specifically for health and mental health. Interestingly, we also found a general increase in the likelihood of using other technologies to support one’s mental health, including gaming and work products, among those experiencing symptoms of mental illness. This increase in the use of other technologies may reflect the transition to working from home that occurred for many people.

We also sought to examine if the likelihood of using these tools and technologies was the highest among people who were both living in counties with high COVID-19 case rates *and* exhibiting symptoms indicative of clinically meaningful levels of depression or anxiety. The findings suggested, in fact, that there was no additional significant likelihood of increased use among respondents with depressive or anxious symptoms living in counties with high COVID-19 case rates compared to individuals with similar symptom levels in counties with low COVID-19 rates. Of course, it is likely that other factors, such as the impacts of policy measures aimed at mitigating risks (eg, physical distancing, working from home, or school closures) may have had a greater impact on mental health symptoms, resulting in people turning to digital tools for mental health support. In the absence of in-person connections or services, digital tools appear to play an important role in combatting the mental health impact of the pandemic.

We also found that the odds of having used these technologies generally increased over time. This increase in use of digital mental health tools and other technologies may be due, in part, to increased access to some of these technologies. For example, cities like New York City [30] have made technologies freely available to their residents and/or shared recommendations for particular products that have been vetted. Additionally, health insurance companies, such as Kaiser Permanente and others, have also made mental health products freely available for their enrollees [31]. It might also be due to the fact that people are spending more time interacting with technologies and these may be convenient ways to receive mental health support.

Our findings that people are increasingly using various technologies—both those specifically designed for mental health support as well as those that are not—are consistent with studies that explore the various ways people use technology to self-manage their mental health [32-34]. These findings also support recent calls for understanding how the technology ecosystem might impact mental health and lead to opportunities for prevention and intervention tools [35]. Some efforts, such as Google’s integration of mental health screening into their search engine, have been launched [36]. In light of our findings, it appears as though there is consumer interest for such resources. Furthermore, an important consideration for future work is to ensure that consumers find effective and safe resources, and have the proper support for using such resources appropriately.

Indeed, despite this increased use and interest, evidence-based and safe resources are rarely available for consumers. One study has suggested that only 2.08% of publicly available psychosocial wellness and stress management mobile apps have published, peer-reviewed evidence of feasibility and/or efficacy [37]. Furthermore, few products provide sufficient information to gauge their safety and privacy [38], and even among those that do, many share information with third parties in ways that might not be disclosed in those policies [39]. Although various efforts have been launched that either evaluate or offer evaluative frameworks for mental health apps (including One Mind PsyberGuide [40], ORCHA [41], and the American Psychiatric Association app evaluation framework [42]), no widely accepted or coordinated effort at regulation and evaluation exists in the United States, despite multiple calls for such models (see National Institutes of Health, National Advisory Work Group [43]). Indeed, better regulation and better access to information at point-of-access could be a significant improvement in helping guide consumers [44], who are demonstrating a clear interest in such resources, and might serve an important need in light of the COVID-19 pandemic.

Several study limitations should be noted. First, this survey was completed by MTurk workers who regularly use the computer, and therefore have the necessary technology, mobility, and digital literacy to participate [45]. This might contribute to the rates of technology use reported and the ability to use technologies—both those designed for mental health and otherwise—to support their mental health. Additionally, it is likely that people with the lowest socioeconomic status, those that are the most isolated, and those with limited access to technology were omitted from the study. These individuals may represent those most affected by COVID-19 and thus may have the greatest mental health needs [46]. Second, the survey was only available in English, and thus the findings may not hold among groups with limited English proficiency who also might be less likely to use these technologies, especially because many are not designed for diverse populations [47]. Third, this study only included people living in the United States, and thus, findings cannot be generalized to other countries [1]. Fourth, there exist a number of scales pertaining to examining the self-reported impact of COVID-19 on mental health [48-51]. These works were primarily published during the development and implementation of this survey, and as such, they were not included in this study. Fifth, there exists the possibility that at least some of the survey responses were bot-generated. To test for the potential influence of bots, we executed post hoc diagnostics previously suggested by Chmielewski and Kucker [52]. Removing those responses suspected to be bot-generated did not substantially change the findings. Thus, we reported on those findings generated from the full analytic sample. However, we also have included the results garnered after removing tagged responses (Multimedia Appendix 4).

Finally, this study focused on examining the extent to which people used mobile health technologies throughout the early period of the pandemic. The findings do not shed light on the effectiveness of these particular strategies for managing mental health. For many, engagement in digital mental health tool and other technology use has been critical for enhancing social connectedness, managing stress and anxiety, and providing greatly needed entertainment [53]. Although the vast majority of the increase in digital technology use is adaptive, it is important to note that there are likely negative impacts of this growth, including the spread of false information [54-56]. Furthermore, there exist subgroups of vulnerable individuals that are at risk of developing problematic usage patterns [53]. Excessive engagement in specific online activities such as video gaming, social media use, and shopping has been linked with severe problems and can elevate the risk of disordered or addictive use [57,58].

In conclusion, this study provides an important description of the prevalence of symptoms of anxiety and depression and digital mental health tool and other technology use during the early stages of the COVID-19 pandemic. Specifically, we found evidence of an increased likelihood of experiencing depressive symptoms both when considering differences between county COVID-19 case rates and changes over time. Furthermore, COVID-19 case rate and time were generally associated with an increased likelihood of using digital mental health tools and other technologies. Lastly, those experiencing symptoms of depression or anxiety were more likely than those without such symptoms to use tools and technologies to manage their mental health.

As the pandemic is expected to continue well into the spring and summer of 2021, and as depression [59,60], anxiety [7,61], and suicidal ideation rates continue to climb [1,8,62], the importance of providing easy access to tools and technologies to manage one’s mental health will become even more important. The current shortage of mental health professionals demands the need to explore more scalable solutions that might be able to be adapted and deployed to meet the needs of various populations. The findings from this study—as well as future research that may address more specific issues designed to understand how digital mental health tools and other technologies can be more accessible and effective for the populations who need them—could inform public health efforts.

## Appendix

Multimedia Appendix 1 Regression models employing full sample.

Multimedia Appendix 2 Predicted probability of using a type of digital mental health tool or other technology at each time point when covariates are at their mean values.

Multimedia Appendix 3 Number of participants using a type of digital mental health tool or other technology at each time point.

Multimedia Appendix 4 Regression models using the sample generated after removing responses suspected to be bot-generated.

## Abbreviations

GAD-7: Generalized Anxiety Disorder-7
MTurk: Amazon Mechanical Turk
OR: odds ratio
PHQ-9: Patient Health Questionnaire-9
