# Supplementary material for: Rise in Use of Digital Mental Health Tools and Technologies in the United States During the COVID-19 Pandemic: Survey Study
Source: J Med Internet Res. 2021 Apr 16;23(4):e26994. doi: 10.2196/26994 (PMC8054774; doi:10.2196/26994)
Supplement: Multimedia Appendix 1 [file jmir_v23i4e26994_app1.docx]

| Table 1. Replication of models showing association between symptoms indicative of clinical levels of depression and anxiety and rates of COVID-19 Cases and time, with all covariates shown | | |
| --- | --- | --- |
|  | Model 1:  Symptoms of Depression  (n=5,899) | Model 2:  Symptoms of Anxiety  (n=5,899) |
|  | OR [95% CI] | OR [95% CI] |
| County-level COVID-19 case rate per ten people | 2.06*  [1.27,3.35] | 1.21  [0.77,1.88] |
| Survey Time Point | 1.19**  [1.12,1.27] | 1.12**  [1.05,1.19] |
| State Fixed Effects (REF=Alabama) |  |  |
| Alaska | 0.89  [0.29,2.73] | 0.64  [0.19,2.16] |
| Arizona | 0.82  [0.44,1.55] | 0.64  [0.33,1.25] |
| Arkansas | 0.58  [0.25,1.31] | 0.69  [0.30,1.57] |
| California | 0.69  [0.42,1.11] | 0.73  [0.43,1.23] |
| Colorado | 0.85  [0.44,1.64] | 0.55  [0.28,1.10] |
| Connecticut | 0.63  [0.29,1.39] | 0.58  [0.25,1.36] |
| Delaware | 0.70  [0.19,2.63] | 0.66  [0.20,2.20] |
| District of Columbia | 0.43  [0.12,1.50] | 0.39  [0.12,1.29] |
| Florida | 0.50^ǂ^  [0.29,0.86] | 0.53^ǂ^  [0.30,0.95] |
| Georgia | 0.56  [0.30,1.04] | 0.62  [0.33,1.18] |
| Hawaii | 0.47  [0.15,1.46] | 0.82  [0.26,2.61] |
| Idaho | 0.69  [0.25,1.94] | 0.94  [0.34,2.63] |
| Illinois | 0.73  [0.42,1.30] | 0.88  [0.48,1.62] |
| Indiana | 0.87  [0.45,1.67] | 0.86  [0.44,1.70] |
| Iowa | 0.32^ǂ^  [0.13,0.78] | 0.30^ǂ^  [0.12,0.76] |
| Kansas | 0.83  [0.36,1.90] | 0.63  [0.28,1.44] |
| Kentucky | 0.44^ǂ^  [0.21,0.94] | 0.88  [0.42,1.84] |
| Louisiana | 0.63  [0.32,1.28] | 0.69  [0.32,1.47] |
| Maine | 0.67  [0.16,2.78] | 0.23  [0.05,1.05] |
| Maryland | 0.91  [0.47,1.77] | 1.38  [0.68,2.79] |
| Massachusetts | 0.30*  [0.15,0.62] | 0.55  [0.27,1.14] |
| Michigan | 0.46^ǂ^  [0.25,0.83] | 0.41^ǂ^  [0.21,0.78] |
| Minnesota | 0.41^ǂ^  [0.19,0.86] | 0.45^ǂ^  [0.21,0.97] |
| Mississippi | 0.69  [0.29,1.63] | 0.64  [0.29,1.42] |
| Missouri | 0.70  [0.37,1.32] | 0.82  [0.42,1.61] |
| Montana | 0.49  [0.13,1.84] | 0.25  [0.04,1.53] |
| Nebraska | 0.31^ǂ^  [0.11,0.85] | 0.66  [0.26,1.69] |
| Nevada | 0.58  [0.27,1.25] | 0.30^ǂ^  [0.13,0.68] |
| New Hampshire | 0.91  [0.29,2.90] | 0.54  [0.16,1.84] |
| New Jersey | 0.65  [0.34,1.26] | 0.78  [0.40,1.55] |
| New Mexico | 0.99  [0.38,2.61] | 1.16  [0.44,3.05] |
| New York | 0.60  [0.34,1.04] | 0.72  [0.39,1.30] |
| North Carolina | 0.43^ǂ^  [0.23,0.78] | 0.54  [0.29,1.02] |
| North Dakota | 0.40  [0.09,1.74] | 0.54  [0.14,2.13] |
| Ohio | 0.59  [0.33,1.07] | 0.55  [0.30,1.02] |
| Oklahoma | 0.75  [0.36,1.56] | 0.67  [0.32,1.40] |
| Oregon | 0.38^ǂ^  [0.18,0.83] | 0.54  [0.25,1.17] |
| Pennsylvania | 0.59  [0.33,1.04] | 0.74  [0.40,1.37] |
| Rhode Island | 0.24  [0.06,1.01] | 0.36  [0.09,1.43] |
| South Carolina | 0.44^ǂ^  [0.20,0.97] | 0.42^ǂ^  [0.20,0.91] |
| South Dakota | 0.18^ǂ^  [0.04,0.84] | 0.67  [0.20,2.30] |
| Tennessee | 0.52  [0.26,1.01] | 0.60  [0.30,1.22] |
| Texas | 0.73  [0.44,1.23] | 0.69  [0.40,1.20] |
| Utah | 0.42^ǂ^  [0.19,0.95] | 0.36^ǂ^  [0.15,0.86] |
| Vermont | 0.28  [0.04,1.83] | 0.16  [0.02,1.30] |
| Virginia | 0.61  [0.33,1.12] | 0.67  [0.35,1.27] |
| Washington | 0.59  [0.32,1.09] | 0.68  [0.36,1.30] |
| West Virginia | 0.58  [0.17,1.98] | 1.00  [0.31,3.19] |
| Wisconsin | 0.63  [0.31,1.26] | 0.69  [0.34,1.40] |
| Wyoming | 0.36  [0.10,1.29] | 0.84  [0.22,3.21] |
| Age | 0.97**  [0.97,0.98] | 0.97**  [0.97,0.98] |
| Sex | 0.85^ǂ^  [0.74,0.96] | 0.88^ǂ^  [0.77,1.00] |
| Race/Ethnicity (REF=non-Hispanic white) |  |  |
| Latino | 2.35**  [1.92,2.88] | 2.10**  [1.73,2.54] |
| Asian | 0.60**  [0.45,0.78] | 0.61**  [0.46,0.81] |
| Black | 0.96  [0.75,1.23] | 1.14  [0.90,1.46] |
| Other | 2.75**  [2.27,3.34] | 2.71**  [2.25,3.26] |
| Missing | 1.43  [0.78,2.61] | 1.62  [0.88,2.98] |
| Income | 0.86**  [0.84,0.88] | 0.88**  [0.86,0.91] |
| Education | 1.86**  [1.55,2.23] | 1.69**  [1.41,2.02] |
| Marital Status (REF=Married/living in a marital-like relationship) |  |  |
| Single/never married | 0.49**  [0.42,0.58] | 0.48**  [0.41,0.56] |
| Separated, divorced, or widowed | 0.61*  [0.45,0.84] | 0.59*  [0.42,0.81] |
| Missing | 0.95  [0.48,1.85] | 1.24  [0.60,2.56] |
| Employment Status (REF=No change in employment status due to COVID-19) |  |  |
| Reduced hours due to COVID-19 | 1.99**  [1.71,2.30] | 1.85**  [1.59,2.14] |
| Lost job due to COVID-19 | 2.10**  [1.65,2.67] | 2.19**  [1.74,2.75] |
| Missing | 2.68**  [2.02,3.54] | 2.41**  [1.84,3.15] |

ǂ *P*<0.05, * *P*<0.0037 (Šidák-corrected p-value), ** *P*<0.001

| Table 2. Replication of models showing associations between Use of Digital Mental Health Tools and Other Technologies and Prevalence of Mental Illness Symptoms and the Rate of COVID-19 Cases, with all covariates shown | | | | | | | |
| --- | --- | --- | --- | --- | --- | --- | --- |
|  | Model 1:  Mental health forums, websites, or apps  (n=5,849) | | Model 2:  Phone-based or text-based crisis lines  (n=5,831) | | Model 3:  Other health forums, websites, or apps  (n=5,854) | | Model 4:  Social Media and Blogs  (n=5,788) |
|  | OR [95% CI] | | OR [95% CI] | | OR [95% CI] | | OR [95% CI] |
| Depressive Symptoms | 6.01**  [4.70,7.70] | | 4.98**  [3.66,6.77] | | 3.44**  [2.81,4.20] | | 1.56**  [1.31,1.86] |
| Anxiety Symptoms | 2.95**  [2.37,3.66] | | 2.85**  [2.22,3.66] | | 2.55**  [2.11,3.10] | | 1.80**  [1.51,2.14] |
| County-level COVID-19 case rate per ten people | 2.70*  [1.49,4.88] | | 1.81^ǂ^  [1.02,3.19] | | 2.60**  [1.55,4.34] | | 1.49  [0.95,2.36] |
| Survey Time Point | 1.20**  [1.11,1.30] | | 1.20**  [1.10,1.31] | | 1.12*  [1.05,1.20] | | 1.08^ǂ^  [1.02,1.15] |
| State Fixed Effects (REF=Alabama) |  | |  | |  | |  |
| Alaska | 2.29  [0.72,7.30] | | 1.47  [0.44,4.88] | | 1.07  [0.37,3.11] | | 1.33  [0.33,5.39] |
| Arizona | 0.80  [0.38,1.69] | | 1.17  [0.51,2.67] | | 0.55  [0.28,1.09] | | 1.29  [0.68,2.45] |
| Arkansas | 0.66  [0.23,1.95] | | 0.84  [0.25,2.76] | | 0.64  [0.23,1.77] | | 1.13  [0.47,2.73] |
| California | 0.86  [0.46,1.59] | | 1.28  [0.66,2.48] | | 0.60  [0.34,1.07] | | 1.20  [0.70,2.03] |
| Colorado | 0.57  [0.24,1.36] | | 1.01  [0.37,2.73] | | 0.63  [0.29,1.34] | | 0.98  [0.48,1.97] |
| Connecticut | 1.26  [0.49,3.24] | | 1.98  [0.64,6.08] | | 0.97  [0.38,2.43] | | 1.43  [0.65,3.14] |
| Delaware | 0.89  [0.18,4.41] | | 0.48  [0.09,2.57] | | 0.67  [0.21,2.14] | | 1.82  [0.56,5.96] |
| District of Columbia | 2.19  [0.52,9.13] | | 6.60*  [2.07,21.10] | | 1.88  [0.52,6.81] | | 1.30  [0.46,3.72] |
| Florida | 0.67  [0.34,1.34] | | 1.38  [0.65,2.94] | | 0.51^ǂ^  [0.27,0.95] | | 1.42  [0.80,2.53] |
| Georgia | 0.74  [0.32,1.68] | | 1.60  [0.65,3.90] | | 0.60  [0.29,1.23] | | 1.32  [0.69,2.50] |
| Hawaii | 1.40  [0.29,6.76] | | 1.27  [0.26,6.28] | | 0.87  [0.25,3.06] | | 1.91  [0.63,5.83] |
| Idaho | 0.15^ǂ^  [0.03,0.71] | | 1.16  [0.31,4.36] | | 0.24^ǂ^  [0.06,0.94] | | 1.14  [0.46,2.85] |
| Illinois | 0.99  [0.47,2.08] | | 0.99  [0.45,2.19] | | 0.70  [0.36,1.39] | | 1.35  [0.73,2.50] |
| Indiana | 1.00  [0.42,2.38] | | 1.25  [0.51,3.06] | | 0.91  [0.40,2.04] | | 1.66  [0.81,3.41] |
| Iowa | 1.13  [0.39,3.27] | | 3.08  [0.95,10.02] | | 1.16  [0.48,2.78] | | 1.21  [0.51,2.85] |
| Kansas | 0.71  [0.20,2.57] | | 0.61  [0.14,2.58] | | 0.42  [0.15,1.18] | | 0.64  [0.25,1.62] |
| Kentucky | 0.43  [0.13,1.39] | | 2.88  [0.90,9.18] | | 0.36^ǂ^  [0.13,0.99] | | 1.19  [0.54,2.60] |
| Louisiana | 1.12  [0.44,2.89] | | 2.56  [0.98,6.65] | | 0.87  [0.37,2.07] | | 1.55  [0.75,3.20] |
| Maine | 0.77  [0.09,6.36] | | . | | 0.13^ǂ^  [0.02,0.78] | | 0.81  [0.26,2.52] |
| Maryland | 1.11  [0.50,2.49] | | 1.4  [0.52,3.77] | | 0.65  [0.29,1.45] | | 1.4  [0.68,2.86] |
| Massachusetts | 0.46  [0.16,1.36] | | 0.62  [0.19,2.07] | | 0.56  [0.24,1.31] | | 1.03  [0.52,2.07] |
| Michigan | 0.63  [0.28,1.43] | | 0.97  [0.40,2.34] | | 0.54  [0.25,1.15] | | 0.82  [0.44,1.55] |
| Minnesota | 0.31  [0.09,1.07] | | 0.69  [0.19,2.45] | | 0.32^ǂ^  [0.13,0.78] | | 0.69  [0.32,1.47] |
| Mississippi | 0.72  [0.23,2.28] | | 2.43  [0.80,7.41] | | 0.74  [0.29,1.93] | | 1.64  [0.73,3.68] |
| Missouri | 0.61  [0.27,1.39] | | 0.79  [0.29,2.14] | | 0.72  [0.34,1.52] | | 1.25  [0.63,2.48] |
| Montana | 1.06  [0.12,9.55] | | . | | 0.17  [0.02,1.66] | | 1.30  [0.31,5.49] |
| Nebraska | 0.96  [0.21,4.40] | | 2.37  [0.91,6.20] | | 0.53  [0.15,1.96] | | 1.39  [0.52,3.72] |
| Nevada | 0.58  [0.24,1.39] | | 1.10  [0.39,3.15] | | 0.49  [0.22,1.12] | | 1.86  [0.83,4.18] |
| New Hampshire | 0.93  [0.12,6.92] | | 1.71  [0.25,11.70] | | 0.94  [0.26,3.48] | | 0.68  [0.21,2.22] |
| New Jersey | 0.83  [0.34,2.04] | | 1.13  [0.44,2.90] | | 1.03  [0.48,2.21] | | 1.17  [0.59,2.34] |
| New Mexico | 0.45  [0.14,1.39] | | 1.83  [0.55,6.10] | | 0.91  [0.26,3.21] | | 0.83  [0.30,2.31] |
| New York | 0.63  [0.30,1.31] | | 1.20  [0.55,2.60] | | 0.49^ǂ^  [0.25,0.94] | | 1.10  [0.61,1.99] |
| North Carolina | 0.68  [0.30,1.52] | | 1.23  [0.50,3.02] | | 0.42^ǂ^  [0.20,0.87] | | 0.99  [0.53,1.86] |
| North Dakota | 0.97  [0.12,8.12] | | 1.77  [0.24,12.97] | | 0.35  [0.04,2.99] | | 1.84  [0.47,7.28] |
| Ohio | 0.76  [0.35,1.66] | | 1.41  [0.61,3.25] | | 0.66  [0.33,1.33] | | 1.18  [0.63,2.22] |
| Oklahoma | 0.85  [0.33,2.20] | | 1.23  [0.45,3.37] | | 0.35^ǂ^  [0.15,0.80] | | 1.20  [0.59,2.43] |
| Oregon | 0.42  [0.14,1.31] | | 1.29  [0.41,4.04] | | 0.45  [0.17,1.18] | | 0.74  [0.33,1.66] |
| Pennsylvania | 0.83  [0.39,1.77] | | 1.60  [0.72,3.57] | | 0.79  [0.40,1.57] | | 1.20  [0.64,2.22] |
| Rhode Island | 1.89  [0.48,7.48] | | 1.45  [0.16,13.29] | | 0.39  [0.07,2.03] | | 0.53  [0.15,1.87] |
| South Carolina | 0.94  [0.37,2.40] | | 2.50  [0.84,7.43] | | 1.07  [0.46,2.50] | | 1.62  [0.77,3.39] |
| South Dakota | 0.43  [0.08,2.40] | | 1.53  [0.07,33.97] | | 0.16^ǂ^  [0.03,0.91] | | 0.39  [0.08,1.86] |
| Tennessee | 0.52  [0.22,1.25] | | 1.53  [0.64,3.65] | | 0.59  [0.27,1.31] | | 1.15  [0.57,2.31] |
| Texas | 0.93  [0.48,1.81] | | 1.11  [0.54,2.31] | | 0.61  [0.33,1.14] | | 1.39  [0.79,2.44] |
| Utah | 0.60  [0.23,1.55] | | 0.92  [0.23,3.66] | | 0.27^ǂ^  [0.09,0.82] | | 0.68  [0.27,1.70] |
| Vermont | 0.70  [0.17,2.93] | | 2.49  [0.57,10.77] | | 1.93  [0.34,10.88] | | 0.50  [0.11,2.28] |
| Virginia | 0.85  [0.39,1.83] | | 1.22  [0.52,2.86] | | 0.75  [0.35,1.60] | | 1.79  [0.92,3.49] |
| Washington | 1.30  [0.57,2.97] | | 1.28  [0.53,3.08] | | 0.53  [0.26,1.08] | | 1.15  [0.60,2.20] |
| West Virginia | 1.39  [0.31,6.18] | | 1.90  [0.41,8.75] | | 1.29  [0.35,4.81] | | 2.11  [0.67,6.68] |
| Wisconsin | 0.82  [0.33,2.00] | | 1.44  [0.56,3.69] | | 0.63  [0.27,1.47] | | 1.39  [0.68,2.86] |
| Wyoming | 0.70  [0.15,3.27] | | 0.44  [0.05,3.57] | | 0.74  [0.27,2.04] | | 0.49  [0.13,1.89] |
| Age | 0.99*  [0.98,0.99] | | 0.99  [0.98,1.00] | | 0.99  [0.99,1.00] | | 0.99*  [0.98,1.00] |
| Sex | 0.56**  [0.47,0.67] | | 0.58**  [0.48,0.70] | | 0.73**  [0.63,0.86] | | 0.93  [0.82,1.06] |
| Race/Ethnicity (REF=non-Hispanic white) |  | |  | |  | |  |
| Latino | 2.05**  [1.60,2.62] | | 1.84**  [1.42,2.38] | | 2.09**  [1.68,2.60] | | 1.56**  [1.27,1.91] |
| Asian | 0.39**  [0.24,0.66] | | 0.31**  [0.15,0.61] | | 0.77  [0.55,1.09] | | 1.45^ǂ^  [1.13,1.87] |
| Black | 1.33  [0.98,1.82] | | 1.20  [0.83,1.72] | | 1.71**  [1.29,2.26] | | 2.11**  [1.65,2.71] |
| Other | 2.52**  [2.00,3.18] | | 2.58**  [2.02,3.28] | | 2.15**  [1.73,2.68] | | 1.72**  [1.43,2.08] |
| Missing | 1.62  [0.82,3.19] | | 2.40^ǂ^  [1.18,4.88] | | 1.41  [0.73,2.71] | | 2.22^ǂ^  [1.22,4.02] |
| Income | 0.96^ǂ^  [0.93,0.99] | | 0.96^ǂ^  [0.92,0.99] | | 0.99  [0.96,1.02] | | 0.97^ǂ^  [0.95,1.00] |
| Education | 4.08**  [2.97,5.62] | | 3.83**  [2.62,5.60] | | 2.93**  [2.29,3.75] | | 1.56**  [1.31,1.86] |
| Marital Status (REF=Married/living in a marital-like relationship) |  | |  | |  | |  |
| Single/never married | 0.34**  [0.28,0.43] | | 0.33**  [0.25,0.42] | | 0.36**  [0.30,0.43] | | 0.57**  [0.49,0.66] |
| Separated, divorced, or widowed | 0.21**  [0.12,0.39] | | 0.23**  [0.11,0.47] | | 0.48**  [0.31,0.73] | | 0.55**  [0.41,0.76] |
| Missing | 0.89  [0.42,1.85] | | 1.00  [0.38,2.61] | | 0.41  [0.17,1.00] | | 1.76  [0.78,4.02] |
| Employment Status (REF=No change in employment status due to COVID-19) |  | |  | |  | |  |
| Reduced hours due to COVID-19 | 1.60**  [1.31,1.96] | | 1.2  [0.97,1.49] | | 1.80**  [1.51,2.13] | | 1.27*  [1.10,1.48] |
| Lost job due to COVID-19 | 1.36^ǂ^  [1.05,1.75] | | 1.36^ǂ^  [1.01,1.84] | | 1.46*  [1.14,1.86] | | 0.99  [0.79,1.24] |
| Missing | 2.07**  [1.49,2.89] | | 1.94**  [1.38,2.72] | | 1.68^ǂ^  [1.18,2.39] | | 1.16  [0.87,1.54] |
|  | |  | |  | |  | |
|  | | Model 5:  Online, computer, or console gaming/video gaming  (n=5,866) | | Model 6:  Online calendar, checklist, planner, Word document, notepad, Google Doc, Spreadsheet, or Google Sheet  (n=5,835) | | Model 7:  Email, texting or messaging software, or video conferencing software  (n=5,849) | |
|  | | OR [95% CI] | | OR [95% CI] | | OR [95% CI] | |
| Depressive Symptoms | | 1.63**  [1.36,1.95] | | 1.91**  [1.60,2.28] | | 1.66**  [1.39,1.98] | |
| Anxiety Symptoms | | 1.67**  [1.40,1.99] | | 2.09**  [1.75,2.50] | | 1.82**  [1.52,2.17] | |
| County-level COVID-19 case rate per ten people | | 1.65^ǂ^  [1.07,2.56] | | 2.04^ǂ^  [1.26,3.30] | | 1.77^ǂ^  [1.09,2.89] | |
| Survey Time Point | | 1.12**  [1.05,1.19] | | 1.08^ǂ^  [1.01,1.15] | | 1.08^ǂ^  [1.02,1.14] | |
| State Fixed Effects (REF=Alabama) | |  | |  | |  | |
| Alaska | | 0.99  [0.31,3.11] | | 2.16  [0.60,7.75] | | 1.43  [0.35,5.77] | |
| Arizona | | 0.76  [0.39,1.50] | | 0.90  [0.46,1.74] | | 0.71  [0.38,1.33] | |
| Arkansas | | 1.20  [0.51,2.81] | | 0.45  [0.18,1.15] | | 0.62  [0.28,1.39] | |
| California | | 1.07  [0.61,1.88] | | 1.06  [0.61,1.86] | | 1.03  [0.61,1.73] | |
| Colorado | | 0.58  [0.28,1.24] | | 0.94  [0.46,1.92] | | 0.76  [0.39,1.51] | |
| Connecticut | | 0.82  [0.36,1.85] | | 1.15  [0.49,2.69] | | 1.14  [0.54,2.44] | |
| Delaware | | 1.78  [0.48,6.61] | | 2.22  [0.43,11.34] | | 8.27  [0.84,81.02] | |
| District of Columbia | | 1.62  [0.54,4.89] | | 1.78  [0.62,5.13] | | 1.58  [0.54,4.60] | |
| Florida | | 0.98  [0.53,1.81] | | 0.93  [0.50,1.71] | | 0.99  [0.56,1.74] | |
| Georgia | | 0.74  [0.37,1.48] | | 0.71  [0.36,1.39] | | 0.73  [0.39,1.39] | |
| Hawaii | | 1.96  [0.76,5.03] | | 1.44  [0.47,4.36] | | 1.07  [0.38,2.96] | |
| Idaho | | 0.30  [0.09,1.06] | | 0.39  [0.11,1.38] | | 0.32  [0.10,1.01] | |
| Illinois | | 1.04  [0.54,1.99] | | 1.12  [0.59,2.14] | | 1.41  [0.76,2.61] | |
| Indiana | | 0.99  [0.47,2.09] | | 0.99  [0.47,2.08] | | 1.32  [0.66,2.67] | |
| Iowa | | 0.95  [0.39,2.34] | | 0.76  [0.33,1.73] | | 1.24  [0.52,2.92] | |
| Kansas | | 0.38  [0.14,1.05] | | 0.70  [0.27,1.82] | | 0.52  [0.21,1.28] | |
| Kentucky | | 0.75  [0.31,1.78] | | 0.94  [0.40,2.21] | | 0.91  [0.43,1.93] | |
| Louisiana | | 0.90  [0.41,1.96] | | 1.34  [0.60,2.98] | | 0.94  [0.45,1.98] | |
| Maine | | 1.00  [0.29,3.44] | | 0.77  [0.22,2.70] | | 0.57  [0.17,1.88] | |
| Maryland | | 0.87  [0.41,1.86] | | 0.69  [0.33,1.45] | | 0.77  [0.38,1.55] | |
| Massachusetts | | 0.88  [0.42,1.85] | | 1.03  [0.50,2.14] | | 0.76  [0.38,1.49] | |
| Michigan | | 0.66  [0.33,1.30] | | 0.67  [0.34,1.30] | | 0.64  [0.34,1.19] | |
| Minnesota | | 0.54  [0.24,1.23] | | 0.56  [0.25,1.25] | | 0.69  [0.34,1.43] | |
| Mississippi | | 1.63  [0.67,3.95] | | 1.01  [0.42,2.43] | | 1.24  [0.54,2.84] | |
| Missouri | | 0.79  [0.39,1.61] | | 0.99  [0.48,2.01] | | 0.95  [0.48,1.85] | |
| Montana | | 0.58  [0.10,3.20] | | 0.68  [0.12,3.89] | | 1.29  [0.29,5.70] | |
| Nebraska | | 0.38  [0.09,1.55] | | 1.52  [0.58,4.00] | | 1.27  [0.49,3.29] | |
| Nevada | | 0.55  [0.24,1.29] | | 1.30  [0.57,2.94] | | 0.87  [0.39,1.92] | |
| New Hampshire | | 0.63  [0.18,2.22] | | 1.11  [0.37,3.38] | | 0.57  [0.18,1.81] | |
| New Jersey | | 0.96  [0.47,1.96] | | 0.73  [0.35,1.50] | | 1.00  [0.51,1.96] | |
| New Mexico | | 0.98  [0.34,2.80] | | 0.65  [0.22,1.98] | | 0.70  [0.25,1.94] | |
| New York | | 0.85  [0.45,1.60] | | 0.77  [0.41,1.43] | | 0.82  [0.46,1.48] | |
| North Carolina | | 0.77  [0.39,1.51] | | 0.80  [0.41,1.54] | | 0.72  [0.39,1.32] | |
| North Dakota | | 0.42  [0.07,2.68] | | 1.04  [0.20,5.30] | | 0.98  [0.23,4.12] | |
| Ohio | | 0.84  [0.43,1.64] | | 0.99  [0.51,1.93] | | 0.93  [0.50,1.73] | |
| Oklahoma | | 1.15  [0.54,2.44] | | 0.66  [0.30,1.43] | | 0.45^ǂ^  [0.22,0.91] | |
| Oregon | | 0.83  [0.37,1.88] | | 0.53  [0.23,1.22] | | 0.65  [0.31,1.35] | |
| Pennsylvania | | 0.96  [0.51,1.84] | | 0.75  [0.39,1.44] | | 0.83  [0.45,1.52] | |
| Rhode Island | | 1.41  [0.38,5.22] | | 1.03  [0.31,3.41] | | 1.05  [0.34,3.26] | |
| South Carolina | | 1.44  [0.64,3.22] | | 0.72  [0.33,1.59] | | 0.88  [0.42,1.85] | |
| South Dakota | | 0.48  [0.08,3.08] | | 0.71  [0.15,3.40] | | 0.35  [0.08,1.53] | |
| Tennessee | | 0.62  [0.29,1.33] | | 0.65  [0.31,1.37] | | 0.79  [0.40,1.57] | |
| Texas | | 0.94  [0.52,1.72] | | 1.20  [0.66,2.16] | | 1.14  [0.66,1.98] | |
| Utah | | 0.75  [0.30,1.89] | | 0.99  [0.40,2.45] | | 0.86  [0.36,2.05] | |
| Vermont | | 0.37  [0.03,4.53] | | 0.23  [0.05,1.14] | | 0.89  [0.23,3.47] | |
| Virginia | | 0.65  [0.31,1.35] | | 1.29  [0.64,2.62] | | 1.17  [0.59,2.31] | |
| Washington | | 0.67  [0.33,1.35] | | 0.85  [0.43,1.68] | | 1.00  [0.53,1.89] | |
| West Virginia | | 0.57  [0.16,2.11] | | 1.07  [0.32,3.54] | | 0.97  [0.32,2.89] | |
| Wisconsin | | 1.06  [0.49,2.33] | | 1.30  [0.61,2.77] | | 1.10  [0.54,2.25] | |
| Wyoming | | 0.77  [0.17,3.58] | | 1.09  [0.29,4.09] | | 1.81  [0.43,7.59] | |
| Age | | 0.99**  [0.98,0.99] | | 0.99^ǂ^  [0.98,1.00] | | 1.00  [0.99,1.00] | |
| Sex | | 0.62**  [0.54,0.71] | | 0.74**  [0.65,0.85] | | 0.78**  [0.69,0.89] | |
| Race/Ethnicity (REF=non-Hispanic white) | |  | |  | |  | |
| Latino | | 1.59**  [1.30,1.94] | | 1.97**  [1.61,2.42] | | 1.58**  [1.29,1.94] | |
| Asian | | 1.32^ǂ^  [1.02,1.71] | | 1.39^ǂ^  [1.06,1.82] | | 1.31^ǂ^  [1.02,1.68] | |
| Black | | 1.20  [0.93,1.53] | | 1.46^ǂ^  [1.13,1.89] | | 1.51*  [1.18,1.94] | |
| Other | | 1.72**  [1.43,2.08] | | 1.88**  [1.54,2.28] | | 1.62**  [1.34,1.97] | |
| Missing | | 1.17  [0.63,2.17] | | 1.81  [0.94,3.50] | | 1.50  [0.79,2.88] | |
| Income | | 0.95**  [0.93,0.98] | | 0.98  [0.96,1.00] | | 0.98  [0.96,1.01] | |
| Education | | 1.05  [0.88,1.26] | | 2.58**  [2.12,3.13] | | 1.63**  [1.37,1.93] | |
| Marital Status (REF=Married/living in a marital-like relationship) | |  | |  | |  | |
| Single/never married | | 0.66**  [0.56,0.77] | | 0.45**  [0.38,0.52] | | 0.56**  [0.49,0.66] | |
| Separated, divorced, or widowed | | 0.49**  [0.34,0.71] | | 0.49**  [0.35,0.68] | | 0.63*  [0.47,0.84] | |
| Missing | | 1.01  [0.47,2.19] | | 1.48  [0.70,3.16] | | 1.44  [0.61,3.38] | |
| Employment Status (REF=No change in employment status due to COVID-19) | |  | |  | |  | |
| Reduced hours due to COVID-19 | | 1.14  [0.98,1.34] | | 1.40**  [1.19,1.63] | | 1.31**  [1.13,1.53] | |
| Lost job due to COVID-19 | | 1.24  [0.98,1.56] | | 0.97  [0.76,1.22] | | 1.01  [0.80,1.26] | |
| Missing | | 1.19  [0.91,1.56] | | 1.09  [0.82,1.45] | | 1.19  [0.90,1.57] | |

ǂ *P*<0.05, * *P*<0.0037 (Šidák-corrected p-value), ** *P*<0.001
