# Supplementary figures and images for: Rise in Use of Digital Mental Health Tools and Technologies in the United States During the COVID-19 Pandemic: Survey Study
Source: J Med Internet Res. 2021 Apr 16;23(4):e26994. doi: 10.2196/26994 (PMC8054774; doi:10.2196/26994)

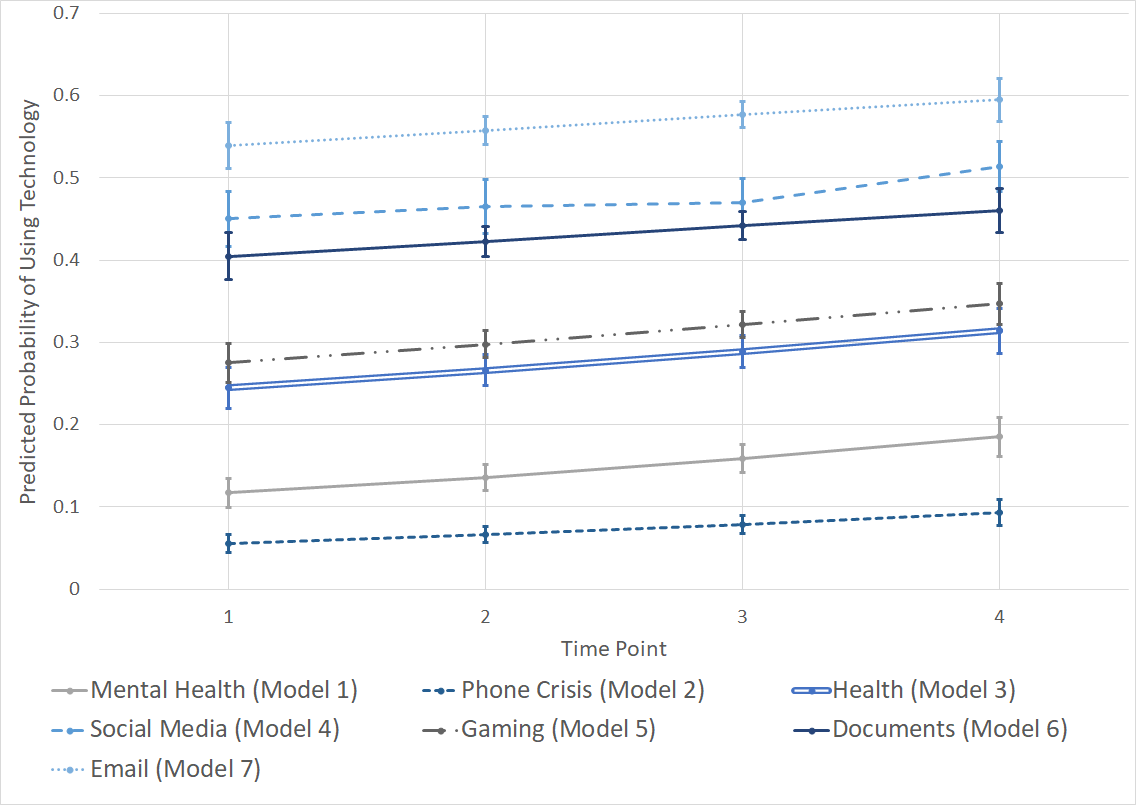

Supplement: Multimedia Appendix 2 [file jmir_v23i4e26994_app2.png]

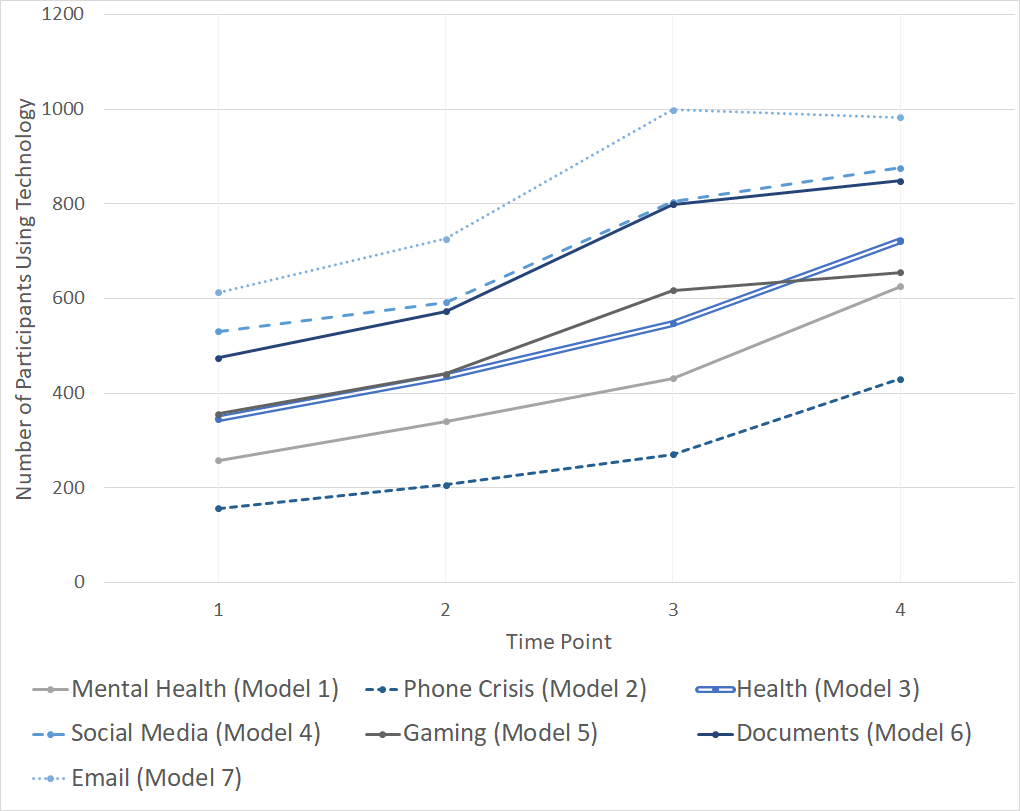

Supplement: Multimedia Appendix 3 [file jmir_v23i4e26994_app3.png]
