# Supplementary material for: Rise in Use of Digital Mental Health Tools and Technologies in the United States During the COVID-19 Pandemic: Survey Study
Source: J Med Internet Res. 2021 Apr 16;23(4):e26994. doi: 10.2196/26994 (PMC8054774; doi:10.2196/26994)
Supplement: Multimedia Appendix 4 [file jmir_v23i4e26994_app4.docx]

| Table 1. Replication of models showing association between symptoms indicative of clinical levels of depression and anxiety and rates of COVID-19 Cases and time, employing smaller sample, with all covariates shown | | |
| --- | --- | --- |
|  | Model 1:  Symptoms of Depression  (n=5,339) | Model 2:  Symptoms of Anxiety  (n=5,339) |
|  | OR [95% CI] | OR [95% CI] |
| County-level COVID-19 case rate per ten people | 2.04^ǂ^  [1.22,3.41] | 1.18  [0.74,1.89] |
| Wave | 1.19**  [1.12,1.27] | 1.13**  [1.06,1.20] |
| State Fixed Effects (REF=Alabama) |  |  |
| Alaska | 0.81  [0.25,2.65] | 0.76  [0.21,2.78] |
| Arizona | 0.83  [0.43,1.60] | 0.70  [0.36,1.40] |
| Arkansas | 0.59  [0.26,1.37] | 0.69  [0.30,1.61] |
| California | 0.67  [0.41,1.10] | 0.72  [0.42,1.23] |
| Colorado | 0.77  [0.39,1.52] | 0.53  [0.26,1.09] |
| Connecticut | 0.64  [0.29,1.41] | 0.59  [0.25,1.40] |
| Delaware | 0.68  [0.17,2.71] | 0.61  [0.17,2.17] |
| District of Columbia | 0.34  [0.08,1.48] | 0.25  [0.06,1.10] |
| Florida | 0.46^ǂ^  [0.26,0.79] | 0.52^ǂ^  [0.29,0.94] |
| Georgia | 0.50^ǂ^  [0.26,0.94] | 0.67  [0.35,1.30] |
| Hawaii | 0.31  [0.09,1.05] | 0.56  [0.16,1.94] |
| Idaho | 0.73  [0.26,2.07] | 1.00  [0.35,2.82] |
| Illinois | 0.62  [0.34,1.11] | 0.80  [0.42,1.51] |
| Indiana | 0.84  [0.43,1.66] | 0.77  [0.38,1.57] |
| Iowa | 0.32^ǂ^  [0.13,0.78] | 0.30^ǂ^  [0.12,0.76] |
| Kansas | 0.78  [0.32,1.87] | 0.64  [0.26,1.53] |
| Kentucky | 0.42^ǂ^  [0.20,0.90] | 0.85  [0.40,1.81] |
| Louisiana | 0.58  [0.28,1.21] | 0.66  [0.30,1.45] |
| Maine | 0.33  [0.07,1.63] | 0.07^ǂ^  [0.01,0.60] |
| Maryland | 0.87  [0.44,1.71] | 1.41  [0.69,2.88] |
| Massachusetts | 0.31*  [0.15,0.65] | 0.54  [0.25,1.15] |
| Michigan | 0.45^ǂ^  [0.24,0.83] | 0.40^ǂ^  [0.21,0.78] |
| Minnesota | 0.36^ǂ^  [0.16,0.79] | 0.41^ǂ^  [0.18,0.93] |
| Mississippi | 0.67  [0.28,1.61] | 0.62  [0.27,1.40] |
| Missouri | 0.64  [0.33,1.24] | 0.83  [0.42,1.66] |
| Montana | 0.51  [0.14,1.85] | 0.26  [0.04,1.59] |
| Nebraska | 0.34^ǂ^  [0.12,0.95] | 0.73  [0.28,1.91] |
| Nevada | 0.55  [0.25,1.22] | 0.28^ǂ^  [0.11,0.67] |
| New Hampshire | 0.88  [0.27,2.88] | 0.47  [0.12,1.84] |
| New Jersey | 0.68  [0.34,1.33] | 0.9  [0.45,1.79] |
| New Mexico | 0.93  [0.35,2.50] | 1.12  [0.42,2.99] |
| New York | 0.51^ǂ^  [0.29,0.90] | 0.64  [0.35,1.18] |
| North Carolina | 0.40*  [0.21,0.74] | 0.53  [0.27,1.01] |
| North Dakota | 0.59  [0.15,2.32] | 0.78  [0.21,2.87] |
| Ohio | 0.60  [0.32,1.10] | 0.59  [0.31,1.12] |
| Oklahoma | 0.79  [0.37,1.67] | 0.71  [0.34,1.50] |
| Oregon | 0.35^ǂ^  [0.15,0.78] | 0.54  [0.24,1.23] |
| Pennsylvania | 0.58  [0.33,1.04] | 0.79  [0.42,1.47] |
| Rhode Island | 0.25  [0.06,1.02] | 0.37  [0.09,1.46] |
| South Carolina | 0.43^ǂ^  [0.19,1.00] | 0.44  [0.19,1.00] |
| South Dakota | 0.20^ǂ^  [0.04,0.98] | 0.78  [0.22,2.77] |
| Tennessee | 0.48^ǂ^  [0.24,0.95] | 0.59  [0.29,1.21] |
| Texas | 0.73  [0.43,1.24] | 0.71  [0.40,1.26] |
| Utah | 0.41^ǂ^  [0.17,0.96] | 0.39^ǂ^  [0.16,0.95] |
| Vermont | 0.30  [0.05,1.84] | 0.16  [0.02,1.38] |
| Virginia | 0.44^ǂ^  [0.23,0.83] | 0.48^ǂ^  [0.24,0.95] |
| Washington | 0.53^ǂ^  [0.28,0.98] | 0.66  [0.34,1.27] |
| West Virginia | 0.63  [0.20,2.03] | 0.88  [0.26,2.96] |
| Wisconsin | 0.61  [0.29,1.26] | 0.65  [0.31,1.39] |
| Wyoming | 0.37  [0.10,1.33] | 0.87  [0.23,3.34] |
| Age | 0.97**  [0.97,0.98] | 0.97**  [0.97,0.98] |
| Sex | 0.90  [0.78,1.03] | 0.89  [0.78,1.02] |
| Race/Ethnicity (REF=non-Hispanic white) |  |  |
| Latino | 2.18**  [1.76,2.71] | 1.94**  [1.58,2.39] |
| Asian | 0.55**  [0.41,0.73] | 0.60**  [0.45,0.80] |
| Black | 0.93  [0.72,1.21] | 1.15  [0.89,1.48] |
| Other | 2.54**  [2.07,3.11] | 2.57**  [2.11,3.13] |
| Missing | 1.12  [0.53,2.37] | 1.38  [0.67,2.82] |
| Income | 0.86**  [0.84,0.88] | 0.89**  [0.87,0.91] |
| Education | 1.80**  [1.49,2.17] | 1.65**  [1.36,1.99] |
| Marital Status (REF=Married/living in a marital-like relationship) |  |  |
| Single/never married | 0.53**  [0.45,0.62] | 0.52**  [0.44,0.61] |
| Separated, divorced, or widowed | 0.64^ǂ^  [0.46,0.89] | 0.62^ǂ^  [0.45,0.87] |
| Missing | 0.81  [0.36,1.82] | 1.53  [0.63,3.69] |
| Employment Status (REF=No change in employment status due to COVID-19) |  |  |
| Reduced hours due to COVID-19 | 2.00**  [1.72,2.34] | 1.90**  [1.63,2.22] |
| Lost job due to COVID-19 | 1.93**  [1.49,2.49] | 2.12**  [1.66,2.71] |
| Missing | 2.69**  [2.02,3.59] | 2.54**  [1.91,3.36] |

ǂ *P*<0.05, * *P*<0.0037 (Šidák-corrected p-value), ** *P*<0.001

| Table 2. Replication of models showing associations between Use of Digital Mental Health Tools and Other Technologies and Prevalence of Mental Illness Symptoms and the Rate of COVID-19 Cases, employing smaller sample, with all covariates shown | | | | | | | |
| --- | --- | --- | --- | --- | --- | --- | --- |
|  | Model 1:  Mental health forums, websites, or apps  (n=5,296) | | Model 2:  Phone-based or text-based crisis lines  (n=5,277) | | Model 3:  Other health forums, websites, or apps  (n=5,284) | | Model 4:  Social Media and Blogs  (n=5,237) |
|  | OR [95% CI] | | OR [95% CI] | | OR [95% CI] | | OR [95% CI] |
| Depressive Symptoms | 6.27**  [4.78,8.21] | | 4.65**  [3.37,6.41] | | 3.28**  [2.65,4.08] | | 1.60**  [1.33,1.93] |
| Anxiety Symptoms | 2.99**  [2.35,3.80] | | 3.10**  [2.37,4.05] | | 2.53**  [2.05,3.11] | | 1.74**  [1.44,2.09] |
| County-level COVID-19 case rate per ten people | 4.02**  [2.11,7.66] | | 2.16^ǂ^  [1.11,4.21] | | 2.89**  [1.66,5.03] | | 1.65^ǂ^  [1.01,2.70] |
| Wave | 1.21**  [1.11,1.32] | | 1.28**  [1.16,1.41] | | 1.13*  [1.04,1.21] | | 1.11**  [1.04,1.18] |
| State Fixed Effects (REF=Alabama) |  | |  | |  | |  |
| Alaska | 3.54^ǂ^  [1.14,10.98] | | 1.06  [0.28,4.03] | | 1.35  [0.47,3.86] | | 1.43  [0.28,7.24] |
| Arizona | 0.93  [0.41,2.10] | | 1.31  [0.53,3.21] | | 0.58  [0.28,1.20] | | 1.25  [0.64,2.44] |
| Arkansas | 0.66  [0.20,2.18] | | 0.99  [0.28,3.48] | | 0.63  [0.22,1.85] | | 1.12  [0.44,2.81] |
| California | 0.93  [0.47,1.83] | | 1.32  [0.65,2.69] | | 0.60  [0.33,1.08] | | 1.21  [0.70,2.10] |
| Colorado | 0.61  [0.23,1.61] | | 1.17  [0.40,3.47] | | 0.62  [0.28,1.40] | | 0.97  [0.46,2.04] |
| Connecticut | 1.43  [0.52,3.91] | | 2.17  [0.65,7.23] | | 1.14  [0.46,2.84] | | 1.48  [0.66,3.34] |
| Delaware | 1.66  [0.39,7.05] | | 0.64  [0.11,3.70] | | 0.62  [0.18,2.15] | | 1.73  [0.51,5.95] |
| District of Columbia | 2.18  [0.44,10.78] | | 5.99^ǂ^  [1.51,23.71] | | 1.72  [0.43,6.88] | | 1.03  [0.32,3.31] |
| Florida | 0.73  [0.34,1.58] | | 1.40  [0.61,3.20] | | 0.51^ǂ^  [0.27,0.99] | | 1.48  [0.81,2.70] |
| Georgia | 0.80  [0.32,2.00] | | 2.07  [0.79,5.44] | | 0.58  [0.27,1.24] | | 1.33  [0.68,2.61] |
| Hawaii | 1.58  [0.26,9.81] | | 0.72  [0.19,2.74] | | 1.02  [0.25,4.18] | | 1.78  [0.54,5.90] |
| Idaho | 0.18^ǂ^  [0.04,0.88] | | 1.39  [0.36,5.39] | | 0.27  [0.07,1.08] | | 1.24  [0.49,3.13] |
| Illinois | 1.10  [0.48,2.49] | | 1.17  [0.49,2.79] | | 0.81  [0.40,1.65] | | 1.46  [0.76,2.77] |
| Indiana | 0.91  [0.34,2.43] | | 1.45  [0.55,3.82] | | 0.96  [0.41,2.27] | | 1.78  [0.84,3.76] |
| Iowa | 1.21  [0.39,3.73] | | 4.31^ǂ^  [1.34,13.84] | | 1.18  [0.48,2.88] | | 1.23  [0.51,2.97] |
| Kansas | 0.63  [0.13,3.02] | | 0.34  [0.04,2.77] | | 0.38  [0.12,1.22] | | 0.47  [0.16,1.38] |
| Kentucky | 0.46  [0.14,1.50] | | 2.61  [0.84,8.12] | | 0.31^ǂ^  [0.11,0.84] | | 1.30  [0.58,2.89] |
| Louisiana | 1.31  [0.45,3.78] | | 2.62  [0.88,7.83] | | 1.00  [0.40,2.50] | | 1.43  [0.67,3.09] |
| Maine | 0.74  [0.06,8.87] | | . | | . | | 0.39  [0.08,1.87] |
| Maryland | 1.23  [0.51,2.93] | | 1.58  [0.55,4.55] | | 0.73  [0.32,1.66] | | 1.67  [0.80,3.49] |
| Massachusetts | 0.55  [0.18,1.74] | | 0.88  [0.26,3.04] | | 0.58  [0.24,1.38] | | 1.13  [0.56,2.32] |
| Michigan | 0.70  [0.28,1.73] | | 1.18  [0.45,3.05] | | 0.57  [0.26,1.24] | | 0.89  [0.46,1.71] |
| Minnesota | 0.19^ǂ^  [0.04,0.84] | | 0.37  [0.07,2.02] | | 0.22*  [0.08,0.58] | | 0.50  [0.21,1.15] |
| Mississippi | 0.95  [0.28,3.20] | | 2.40  [0.71,8.12] | | 0.73  [0.27,1.98] | | 1.66  [0.72,3.81] |
| Missouri | 0.63  [0.25,1.57] | | 0.79  [0.25,2.43] | | 0.70  [0.31,1.57] | | 1.29  [0.63,2.65] |
| Montana | 1.32  [0.14,12.55] | | . | | 0.19  [0.02,1.82] | | 1.37  [0.32,5.81] |
| Nebraska | 1.11  [0.22,5.55] | | 2.64  [0.98,7.14] | | 0.56  [0.15,2.12] | | 1.53  [0.55,4.25] |
| Nevada | 0.56  [0.22,1.44] | | 1.49  [0.48,4.68] | | 0.50  [0.21,1.18] | | 2.25  [0.99,5.13] |
| New Hampshire | 1.85  [0.37,9.37] | | 3.28  [0.57,18.78] | | 0.96  [0.24,3.82] | | 0.62  [0.16,2.32] |
| New Jersey | 0.78  [0.29,2.08] | | 0.88  [0.31,2.46] | | 0.97  [0.44,2.16] | | 1.34  [0.66,2.72] |
| New Mexico | 0.44  [0.12,1.54] | | 2.67  [0.81,8.88] | | 0.94  [0.26,3.44] | | 0.82  [0.28,2.42] |
| New York | 0.57  [0.25,1.30] | | 1.13  [0.48,2.65] | | 0.44^ǂ^  [0.22,0.89] | | 1.14  [0.61,2.12] |
| North Carolina | 0.57  [0.23,1.41] | | 1.49  [0.58,3.80] | | 0.41^ǂ^  [0.19,0.88] | | 0.97  [0.50,1.88] |
| North Dakota | 0.55  [0.05,6.10] | | 2.33  [0.23,23.31] | | 0.22  [0.02,2.42] | | 1.61  [0.37,7.11] |
| Ohio | 0.67  [0.28,1.59] | | 1.35  [0.54,3.38] | | 0.54  [0.26,1.13] | | 1.10  [0.57,2.13] |
| Oklahoma | 0.99  [0.36,2.71] | | 1.58  [0.56,4.46] | | 0.37^ǂ^  [0.16,0.86] | | 1.26  [0.61,2.62] |
| Oregon | 0.28  [0.08,1.02] | | 1.39  [0.40,4.88] | | 0.38  [0.13,1.07] | | 0.82  [0.35,1.90] |
| Pennsylvania | 0.83  [0.37,1.89] | | 1.74  [0.73,4.13] | | 0.77  [0.38,1.58] | | 1.20  [0.63,2.27] |
| Rhode Island | 2.27  [0.55,9.39] | | 1.74  [0.20,15.39] | | 0.42  [0.08,2.18] | | 0.56  [0.16,2.00] |
| South Carolina | 0.83  [0.30,2.29] | | 3.21  [1.00,10.31] | | 0.91  [0.37,2.24] | | 1.55  [0.71,3.39] |
| South Dakota | 0.53  [0.09,3.04] | | 1.79  [0.08,40.29] | | 0.18  [0.03,1.03] | | 0.44  [0.09,2.13] |
| Tennessee | 0.55  [0.21,1.45] | | 1.51  [0.58,3.96] | | 0.57  [0.24,1.33] | | 1.16  [0.56,2.42] |
| Texas | 0.98  [0.47,2.03] | | 1.28  [0.58,2.79] | | 0.66  [0.35,1.26] | | 1.52  [0.85,2.72] |
| Utah | 0.74  [0.26,2.09] | | 0.94  [0.23,3.84] | | 0.23^ǂ^  [0.08,0.70] | | 0.77  [0.30,2.00] |
| Vermont | 0.82  [0.19,3.51] | | 2.79  [0.64,12.17] | | 2.03  [0.36,11.39] | | 0.53  [0.11,2.42] |
| Virginia | 0.87  [0.36,2.12] | | 1.56  [0.58,4.22] | | 0.69  [0.30,1.59] | | 2.07^ǂ^  [1.02,4.18] |
| Washington | 1.52  [0.63,3.67] | | 1.42  [0.55,3.67] | | 0.55  [0.26,1.15] | | 1.22  [0.62,2.39] |
| West Virginia | 1.33  [0.22,8.08] | | 1.17  [0.27,4.95] | | 1.05  [0.24,4.67] | | 2.83  [0.82,9.82] |
| Wisconsin | 0.93  [0.35,2.46] | | 1.66  [0.60,4.61] | | 0.75  [0.31,1.79] | | 1.47  [0.69,3.11] |
| Wyoming | 0.78  [0.16,3.83] | | 0.47  [0.06,3.99] | | 0.79  [0.28,2.21] | | 0.51  [0.13,2.01] |
| Age | 0.99*  [0.98,1.00] | | 0.99  [0.98,1.00] | | 0.99^ǂ^  [0.98,1.00] | | 0.99*  [0.98,1.00] |
| Sex | 0.59**  [0.48,0.71] | | 0.59**  [0.48,0.73] | | 0.76*  [0.65,0.90] | | 0.92  [0.81,1.06] |
| Race/Ethnicity (REF=non-Hispanic white) |  | |  | |  | |  |
| Latino | 2.04**  [1.55,2.68] | | 1.81**  [1.34,2.43] | | 2.12**  [1.67,2.69] | | 1.57**  [1.27,1.95] |
| Asian | 0.38**  [0.22,0.65] | | 0.32^ǂ^  [0.15,0.69] | | 0.76  [0.53,1.09] | | 1.45^ǂ^  [1.12,1.89] |
| Black | 1.36  [0.96,1.92] | | 1.18  [0.80,1.74] | | 1.78**  [1.32,2.40] | | 2.21**  [1.70,2.87] |
| Other | 2.81**  [2.18,3.61] | | 2.76**  [2.12,3.60] | | 2.34**  [1.85,2.95] | | 1.78**  [1.45,2.18] |
| Missing | 2.17  [0.95,4.94] | | 2.67^ǂ^  [1.05,6.79] | | 1.67  [0.82,3.37] | | 2.24^ǂ^  [1.13,4.45] |
| Income | 0.96^ǂ^  [0.93,1.00] | | 0.95^ǂ^  [0.92,0.99] | | 0.99  [0.96,1.02] | | 0.98^ǂ^  [0.95,1.00] |
| Education | 4.71**  [3.24,6.85] | | 5.26**  [3.28,8.43] | | 3.03**  [2.31,3.98] | | 1.51**  [1.26,1.81] |
| Marital Status (REF=Married/living in a marital-like relationship) |  | |  | |  | |  |
| Single/never married | 0.30**  [0.24,0.38] | | 0.28**  [0.21,0.37] | | 0.33**  [0.27,0.41] | | 0.53**  [0.45,0.62] |
| Separated, divorced, or widowed | 0.20**  [0.10,0.39] | | 0.11**  [0.04,0.32] | | 0.47*  [0.30,0.74] | | 0.53**  [0.39,0.74] |
| Missing | 1.00  [0.37,2.66] | | 1.67  [0.57,4.90] | | 0.48  [0.19,1.22] | | 1.72  [0.68,4.35] |
| Employment Status (REF=No change in employment status due to COVID-19) |  | |  | |  | |  |
| Reduced hours due to COVID-19 | 1.60**  [1.28,1.99] | | 1.17  [0.92,1.48] | | 1.76**  [1.46,2.11] | | 1.30**  [1.11,1.53] |
| Lost job due to COVID-19 | 1.25  [0.93,1.68] | | 1.26  [0.88,1.80] | | 1.38^ǂ^  [1.05,1.81] | | 0.94  [0.74,1.21] |
| Missing | 2.40**  [1.67,3.47] | | 2.07**  [1.42,3.02] | | 1.84*  [1.26,2.68] | | 1.3  [0.96,1.75] |
|  |  | |  | |  | |  |
|  | | Model 5:  Online, computer, or console gaming/video gaming  (n=5,310) | | Model 6:  Online calendar, checklist, planner, Word document, notepad, Google Doc, Spreadsheet, or Google Sheet  (n=5,279) | | Model 7:  Email, texting or messaging software, or video conferencing software  (n=5,297) | |
|  | | OR [95% CI] | | OR [95% CI] | | OR [95% CI] | |
| Depressive Symptoms | | 1.55**  [1.29,1.88] | | 1.98**  [1.64,2.39] | | 1.66**  [1.38,2.00] | |
| Anxiety Symptoms | | 1.77**  [1.47,2.14] | | 1.94**  [1.61,2.34] | | 1.75**  [1.45,2.11] | |
| County-level COVID-19 case rate per ten people | | 1.91^ǂ^  [1.19,3.05] | | 2.19*  [1.31,3.67] | | 1.72^ǂ^  [1.03,2.89] | |
| Wave | | 1.13**  [1.06,1.20] | | 1.08^ǂ^  [1.01,1.15] | | 1.09^ǂ^  [1.03,1.16] | |
| State Fixed Effects (REF=Alabama) | |  | |  | |  | |
| Alaska | | 1.03  [0.29,3.66] | | 3.03  [0.78,11.81] | | 1.89  [0.40,8.96] | |
| Arizona | | 0.74  [0.36,1.50] | | 1.01  [0.50,2.02] | | 0.76  [0.39,1.45] | |
| Arkansas | | 1.18  [0.49,2.85] | | 0.45  [0.17,1.17] | | 0.59  [0.26,1.35] | |
| California | | 1.09  [0.60,1.97] | | 1.05  [0.59,1.87] | | 1.06  [0.62,1.80] | |
| Colorado | | 0.66  [0.30,1.47] | | 1.05  [0.49,2.26] | | 0.83  [0.41,1.70] | |
| Connecticut | | 0.82  [0.35,1.93] | | 1.34  [0.57,3.15] | | 1.19  [0.55,2.58] | |
| Delaware | | 2.36  [0.61,9.24] | | 3.54  [0.68,18.55] | | 8.35  [0.82,84.76] | |
| District of Columbia | | 1.31  [0.37,4.69] | | 1.57  [0.50,4.91] | | 1.34  [0.43,4.22] | |
| Florida | | 1.00  [0.52,1.90] | | 0.98  [0.52,1.86] | | 1.00  [0.56,1.78] | |
| Georgia | | 0.69  [0.32,1.46] | | 0.73  [0.36,1.50] | | 0.77  [0.39,1.50] | |
| Hawaii | | 1.58  [0.57,4.36] | | 1.33  [0.40,4.47] | | 0.97  [0.33,2.83] | |
| Idaho | | 0.32  [0.09,1.12] | | 0.43  [0.12,1.54] | | 0.34  [0.11,1.08] | |
| Illinois | | 1.18  [0.60,2.32] | | 1.23  [0.63,2.40] | | 1.43  [0.76,2.69] | |
| Indiana | | 1.05  [0.48,2.31] | | 1.08  [0.50,2.36] | | 1.50  [0.73,3.10] | |
| Iowa | | 1.08  [0.44,2.66] | | 0.76  [0.33,1.79] | | 1.26  [0.53,3.02] | |
| Kansas | | 0.34  [0.11,1.11] | | 0.56  [0.19,1.66] | | 0.43  [0.16,1.17] | |
| Kentucky | | 0.77  [0.32,1.89] | | 0.94  [0.40,2.24] | | 1.00  [0.47,2.14] | |
| Louisiana | | 0.92  [0.40,2.13] | | 1.41  [0.60,3.30] | | 1.05  [0.49,2.28] | |
| Maine | | 1.19  [0.29,4.81] | | 0.63  [0.14,2.91] | | 0.71  [0.19,2.66] | |
| Maryland | | 0.98  [0.45,2.13] | | 0.76  [0.35,1.64] | | 0.85  [0.42,1.75] | |
| Massachusetts | | 0.92  [0.43,2.00] | | 1.18  [0.55,2.52] | | 0.83  [0.41,1.67] | |
| Michigan | | 0.65  [0.32,1.33] | | 0.71  [0.35,1.43] | | 0.66  [0.35,1.25] | |
| Minnesota | | 0.47  [0.19,1.16] | | 0.44  [0.19,1.05] | | 0.59  [0.27,1.26] | |
| Mississippi | | 1.64  [0.66,4.11] | | 1.03  [0.42,2.56] | | 1.27  [0.54,2.95] | |
| Missouri | | 0.69  [0.32,1.49] | | 1.07  [0.51,2.24] | | 0.95  [0.47,1.91] | |
| Montana | | 0.63  [0.11,3.62] | | 0.74  [0.13,4.22] | | 1.36  [0.31,5.90] | |
| Nebraska | | 0.41  [0.10,1.72] | | 1.67  [0.62,4.49] | | 1.43  [0.53,3.84] | |
| Nevada | | 0.57  [0.23,1.39] | | 1.41  [0.61,3.26] | | 0.92  [0.41,2.09] | |
| New Hampshire | | 0.77  [0.23,2.62] | | 1.12  [0.35,3.58] | | 0.68  [0.22,2.10] | |
| New Jersey | | 0.99  [0.47,2.09] | | 0.77  [0.36,1.62] | | 1.01  [0.50,2.03] | |
| New Mexico | | 1.2  [0.42,3.46] | | 0.67  [0.21,2.15] | | 0.71  [0.25,2.04] | |
| New York | | 0.79  [0.41,1.55] | | 0.77  [0.40,1.48] | | 0.85  [0.47,1.56] | |
| North Carolina | | 0.79  [0.39,1.59] | | 0.8  [0.41,1.59] | | 0.70  [0.37,1.31] | |
| North Dakota | | 0.50  [0.07,3.37] | | 1.58  [0.31,8.13] | | 1.47  [0.33,6.50] | |
| Ohio | | 0.76  [0.37,1.55] | | 0.95  [0.48,1.91] | | 0.89  [0.47,1.69] | |
| Oklahoma | | 1.2  [0.55,2.61] | | 0.70  [0.32,1.56] | | 0.47^ǂ^  [0.23,0.97] | |
| Oregon | | 0.92  [0.40,2.11] | | 0.54  [0.22,1.30] | | 0.68  [0.32,1.46] | |
| Pennsylvania | | 0.99  [0.51,1.95] | | 0.77  [0.39,1.51] | | 0.88  [0.47,1.64] | |
| Rhode Island | | 1.50  [0.40,5.60] | | 1.11  [0.33,3.72] | | 1.10  [0.35,3.45] | |
| South Carolina | | 1.43  [0.60,3.42] | | 0.70  [0.30,1.63] | | 0.84  [0.39,1.84] | |
| South Dakota | | 0.54  [0.08,3.67] | | 0.81  [0.17,3.95] | | 0.39  [0.09,1.74] | |
| Tennessee | | 0.62  [0.28,1.39] | | 0.61  [0.27,1.34] | | 0.74  [0.36,1.50] | |
| Texas | | 0.98  [0.53,1.84] | | 1.21  [0.65,2.23] | | 1.21  [0.69,2.13] | |
| Utah | | 0.92  [0.36,2.34] | | 1.06  [0.42,2.68] | | 0.91  [0.37,2.22] | |
| Vermont | | 0.39  [0.03,4.78] | | 0.24  [0.05,1.23] | | 0.94  [0.24,3.67] | |
| Virginia | | 0.90  [0.42,1.93] | | 1.46  [0.69,3.09] | | 1.42  [0.70,2.88] | |
| Washington | | 0.72  [0.35,1.51] | | 0.87  [0.43,1.76] | | 1.06  [0.55,2.03] | |
| West Virginia | | 0.77  [0.21,2.83] | | 1.29  [0.34,4.91] | | 1.25  [0.39,4.02] | |
| Wisconsin | | 1.16  [0.51,2.64] | | 1.21  [0.55,2.68] | | 1.03  [0.49,2.15] | |
| Wyoming | | 0.83  [0.18,3.88] | | 1.20  [0.32,4.55] | | 1.93  [0.46,8.10] | |
| Age | | 0.98**  [0.98,0.99] | | 0.99^ǂ^  [0.98,1.00] | | 0.99  [0.99,1.00] | |
| Sex | | 0.62**  [0.54,0.72] | | 0.75**  [0.65,0.86] | | 0.78**  [0.69,0.90] | |
| Race/Ethnicity (REF=non-Hispanic white) | |  | |  | |  | |
| Latino | | 1.51**  [1.22,1.87] | | 2.00**  [1.61,2.49] | | 1.60**  [1.29,1.99] | |
| Asian | | 1.28  [0.98,1.69] | | 1.49^ǂ^  [1.12,1.96] | | 1.31^ǂ^  [1.01,1.70] | |
| Black | | 1.21  [0.94,1.57] | | 1.53*  [1.17,2.00] | | 1.51*  [1.17,1.96] | |
| Other | | 1.81**  [1.48,2.21] | | 1.94**  [1.58,2.39] | | 1.67**  [1.36,2.05] | |
| Missing | | 1.40  [0.66,2.96] | | 2.83^ǂ^  [1.34,5.99] | | 1.92  [0.91,4.03] | |
| Income | | 0.96*  [0.94,0.98] | | 0.99  [0.96,1.01] | | 0.99  [0.96,1.01] | |
| Education | | 0.99  [0.82,1.19] | | 2.48**  [2.02,3.04] | | 1.52**  [1.28,1.82] | |
| Marital Status (REF=Married/living in a marital-like relationship) | |  | |  | |  | |
| Single/never married | | 0.63**  [0.53,0.75] | | 0.43**  [0.37,0.51] | | 0.54**  [0.46,0.63] | |
| Separated, divorced, or widowed | | 0.50**  [0.34,0.73] | | 0.49**  [0.34,0.70] | | 0.62*  [0.46,0.84] | |
| Missing | | 1.32  [0.54,3.20] | | 1.46  [0.63,3.40] | | 2.5  [0.97,6.43] | |
| Employment Status (REF=No change in employment status due to COVID-19) | |  | |  | |  | |
| Reduced hours due to COVID-19 | | 1.12  [0.95,1.32] | | 1.35**  [1.15,1.60] | | 1.32**  [1.12,1.54] | |
| Lost job due to COVID-19 | | 1.32^ǂ^  [1.03,1.70] | | 0.93  [0.72,1.20] | | 0.97  [0.76,1.24] | |
| Missing | | 1.28  [0.97,1.71] | | 1.14  [0.84,1.53] | | 1.29  [0.96,1.72] | |

ǂ *P*<0.05, * *P*<0.0037 (Šidák-corrected p-value), ** *P*<0.001
